# Experience of sexual recovery after percutaneous coronary intervention in young and middle-aged men: a qualitative study

**DOI:** 10.3389/fpubh.2026.1876557

**Published:** 2026-08-13

**Authors:** Yue Mao, Yang Zhan, Daoxin Hua, Rong Li, Mei Li

**Affiliations:** Department of Nursing, The Second Affiliated Hospital of Zhejiang University School of Medicine, Hangzhou, China

**Keywords:** men, percutaneous coronary intervention, qualitative study, Roy adaptation model, sexual recovery

## Abstract

**Background:**

Sexual recovery is an important but often neglected component of cardiac rehabilitation after percutaneous coronary intervention (PCI). Young and middle-aged men may experience concerns about physical safety, sexual confidence, partner relationship, and cultural taboos. However, evidence on sexual recovery after PCI in the Chinese cultural context remains limited.

**Objective:**

This study explored the experiences of sexual recovery among young and middle-aged Chinese men after PCI, guided by the Roy Adaptation Model (RAM).

**Methods:**

This descriptive qualitative study recruited 12 young and middle-aged men after PCI through purposive sampling. Data were collected through face-to-face, semi-structured interviews and analyzed using directed thematic analysis guided by the RAM. The study was reported in accordance with the Consolidated Criteria for Reporting Qualitative Research (COREQ).

**Results:**

Twelve participants completed the interviews. Four key themes were identified and interpreted in relation to the adaptive modes of the Roy Adaptation Model: (1) physical manifestations, including rare discomfort symptoms; (2) sense of control, including worrying about recurrence, shifting to planned and careful sex, and rebuilding a sense of control through self-management; (3) partner relationship, including relationship patterns and supportive adaptation; and (4) sexual guidance, including no clear guidance and the need for private, professional support.

**Conclusions:**

Sexual recovery after PCI among young and middle-aged men is a multidimensional adaptation process involving physical, psychological, relational, and informational aspects. These findings highlight the need to incorporate culturally sensitive, partner-centered sexual health education into after PCI cardiac rehabilitation.

## 1 Introduction

In recent years, coronary heart disease (CHD) has shown an increasing trend toward younger onset worldwide. Percutaneous coronary intervention (PCI) is currently widely used as a major revascularization strategy for CHD (1), yet postoperative quality of life remains a crucial concern. Sexual quality of life is an integral, but frequently neglected, component of health-related quality of life in patients following PCI (2). Sexual health after PCI affects patients' subjective wellbeing, marital intimacy, and overall quality of life; for young and middle-aged patients, it may also influence family functioning and reproductive planning. After a cardiac event, patients often experience ambivalence regarding sexual recovery, they desire to return to a normal sexual life while fearing that sexual activity may precipitate recurrent ischemia, arrhythmias, or even sudden cardiac death (3). Such ambivalence may lead to prolonged sexual avoidance, hinder psychological adjustment, and strain marital or partner relationship. Data indicate that many patients do not return to their pre-event level of sexual activity after PCI or myocardial infarction, and that nearly half remain sexually inactive for several months (4), and difficulties in sexual recovery are also reported more frequently in men than in women (5), despite guideline recommendations supporting sexual recovery in clinically stable patients (6, 7). However, existing evidence suggests that, in patients with clinically stable coronary artery disease, sexual activity generally corresponds to light-to-moderate physical exertion and causes only modest, transient increases in heart rate, blood pressure, and myocardial oxygen demand (8, 9). The absolute excess risk of triggering acute myocardial infarction through sexual activity is very low, estimated at approximately 1 additional event per 10,000 person-years (8). Furthermore, regular sexual activity, such as at least once per week, may also be associated with improved long-term survival (10, 11). Therefore, sexual activity is generally safe for clinically stable PCI patients, and that sexual recovery should be considered an integral component of cardiac rehabilitation (12).

A particular concern is that sexual issues are commonly perceived as sensitive and taboo, and formal sexual counseling is rarely incorporated into routine cardiac care. Even in Western countries, only a minority of patients receive systematic guidance on sexual recovery after an acute coronary event (13). In mainland China, where cultural norms remain relatively conservative, the physician-centered communication model may further widen the mismatch between patients' need for sexual health services after PCI and the availability of professional sexual education (14, 15). In a survey of 88 cardiac nurses, 94.8% reported that they had never been asked by patients about sexual recovery, and 91.4% had never provided related guidance as part of routine education; only 19.0% reported willingness to provide detailed answers, whereas the majority offered vague responses or avoided the topic altogether (14). These proportions are markedly lower than those reported in international studies (16, 17). Existing research on post-PCI sexual issues has mainly focused on estimating the prevalence of sexual dysfunction or describing patients' general informational needs after PCI (15, 18). Much less is known about how young and middle-aged men navigate the process of sexual recovery—how they interpret bodily changes, reconstruct their sexual self concept, renegotiate partner roles, and cope with cultural taboos in the context of chronic coronary artery disease. In particular, there is a lack of in-depth qualitative research capturing patients' lived experiences of adaptation at physical, psychological, and relational levels. This knowledge gap constrains the development of theory-driven, culturally tailored interventions for young and middle-aged Chinese PCI patients.

The Roy Adaptation Model (RAM) conceptualizes individuals as adaptive systems that respond to internal and external stimuli through four adaptive modes: physiological, self concept, role function, and interdependence, and emphasizes multidimensional adaptation in the face of health challenges (19). RAM has been applied to studies of sexual health and chronic illness in other populations (20, 21). Guided by the RAM, this study explored the adaptation process of sexual recovery in young and middle-aged Chinese men after PCI. Specifically, we sought to describe patients' perceived needs, key stimuli, and coping strategies during sexual recovery, and to identify patterns of effective and ineffective adaptation in order to provide a theoretical and empirical basis for developing culturally appropriate, partner-centered sexual health education and rehabilitation nursing programmes for this population.

## 2 Methods

### 2.1 Design

This study employed a qualitative descriptive design informed by the RAM. Semi-structured, face-to-face interviews were conducted with young and middle-aged men after PCI at a tertiary hospital in Zhejiang Province, China, between August and November 2025. In this study, the RAM served as a sensitizing framework to identify key domains of adaptation, inform the development of the interview guide, and support the interpretation of participants' experiences. The study was reported in accordance with the Consolidated Criteria for Reporting Qualitative Research (COREQ) (Supplementary file 1).

### 2.2 Participants and recruitment

Purposive sampling was used to recruit young and middle-aged men who underwent PCI. In this study, young and middle-aged men were defined as those aged 18–59 years. Participants were recruited from the cardiology department of a tertiary hospital in Zhejiang Province, China. Participants were eligible if they: (1) were men aged 18–59 years; (2) were at least 1 month after PCI; (3) had engaged in sexual activity prior to PCI; (4) had experience or attempts related to sexual activity after PCI; (5) were willing to participate and provided written informed consent. Participants were excluded if they had: (1) severe cognitive impairment or communication difficulties that precluded participation in an interview; (2) severe physical or psychiatric conditions that made participation infeasible.

Potential participants were identified through the hospital's electronic medical record system by two members of the research team (ZY and HDX), both of whom were nurses; ZY held a Master's degree in nursing, and HDX held a Bachelor's degree in nursing. The recruiting nurses were not responsible for the participants' current clinical care at the time of recruitment, and some participants had not been directly cared for by them. Participants were informed that participation or refusal would not affect their medical care. Eligible individuals were contacted by telephone, informed about the purpose and procedures of the study, and invited to participate. For participants' convenience, interviews were conducted in participants' free time after their routine outpatient follow-up visits and separately from medical consultations, in a private hospital room without non-participants present. A total of 12 participants were included.

### 2.3 Data collection

Data were collected through semi-structured, face-to-face interviews. All interviews were conducted by the first author (MY), a male manager with a Master's degree in nursing. All members of the research team had been trained in qualitative research methods, including semi-structured interviewing, qualitative coding, and thematic analysis. Before data collection, the interviewer and coders discussed interview techniques, probing strategies, coding procedures, and reflexivity. MY was not involved in the participants' routine clinical care. Before each interview, participants were informed of the purpose and procedures of the study, assured that the interview data would be used for research purposes only, and encouraged to speak openly about their experiences. With participants' permission, the interviews were audio-recorded and transcribed verbatim. Field notes were also taken to document non-verbal cues, such as facial expressions and body language, as well as the interviewer's reflections. The interviews lasted approximately 20–80 min. The duration of the interviews varied according to participants' communication style, willingness to elaborate, and the depth of their experiences. All key topics in the interview guide were covered, with probing questions used when appropriate.

The interview guide was informed by the RAM and included questions on four areas: physiological responses, self-concept, role function, and interdependence. The guide was further refined through discussions among the research team. Interviews began with broad, open-ended questions, followed by flexible prompts to explore participants' physical, psychological, relational, and support-related experiences of sexual recovery after PCI. The semi-structured interview guide is shown in Table 1. Prior to formal data collection, two pilot interviews were conducted with eligible participants to assess the clarity, sensitivity, and sequencing of the questions. Minor revisions were subsequently made to the wording and order of several questions, and the pilot interview data were not included in the final analysis. Data collection proceeded concurrently with preliminary analysis and continued until thematic saturation was reached, defined as the point at which no new significant information relevant to the research question emerged. All invited participants completed the study. No repeat interviews were conducted, and no participants withdrew during the study.

**Table 1:** Semi-structured research guide.

Domain	Questions
Opening question	1. Please describe your experience of resuming sexual activity after PCI
Prompts	When did you first think about resuming sexual activity? Had you resumed it, attempted to resume it, or considered it but not acted on it? What was that experience like?
Physiological	2. What physical changes, symptoms, or concerns did you experience in relation to sexual activity after PCI? 3. How did your physical condition influence your willingness or ability to resume sexual activity?
Prompts	Chest discomfort? Palpitations? Shortness of breath? Fatigue? Reduced stamina? Concerns about safety? Erectile difficulties?
Self-concept	4. How did PCI affect your thoughts and feelings about resuming sexual activity? 5. What psychological burdens or concerns did you experience, and how did you cope with them?
Prompts	Fear of recurrence? Anxiety? Lack of confidence? Uncertainty about safety? Embarrassment? Feeling different from before?
Role-function	6. How did PCI affect intimacy and your role within the relationship with your partner? 7. How did you and your partner respond to changes in sexual activity after PCI?
Prompts	Changes in expectations? Feeling unable to fulfill a partner role? Sense of responsibility? Concerns about sexual performance?
Interdependence	8. What support or guidance did you receive from your partner, family members, or healthcare professionals regarding sexual recovery after PCI? 9. What information or professional guidance did you feel you needed most?
Prompts	Sexual counseling? Discharge education? Timing of guidance? Content of guidance? Unmet informational needs?
Closing question	10. Is there anything else you would like to share about your experience of sexual recovery after PCI?

### 2.4 Data analysis

The interview data were analyzed using directed thematic analysis. Data collection and analysis proceeded concurrently to allow for iterative refinement of the inquiry. Audio-recordings were transcribed verbatim within 24 h of each interview. Transcripts were imported into NVivo 15 to facilitate data management and coding. Two researchers (ZY and HDX) independently read the transcripts repeatedly to achieve immersion and coded meaningful segments related to participants' experiences of sexual recovery after PCI. Relevant data were then assigned to the predetermined RAM categories according to the primary adaptive process reflected in each segment. The coders then compared their coding decisions, with discrepancies resolved through discussion until a consensus was reached. Within each predetermined RAM category, codes were compared iteratively and grouped according to similarities, differences, and patterns, forming subthemes that captured participants' experiences within that category. In this study, stimuli were used to refer to the physical, psychological, relational, and informational factors that participants perceived as influencing sexual recovery after PCI. Adaptive responses referred to participants' ways of responding to these stimuli, including cautious self-regulation, self-management, partner communication, role adjustment, information seeking, and requests for professional support.

### 2.5 Ethical considerations

This study was approved by the Ethics Committee of the Second Affiliated Hospital, Zhejiang University School of Medicine [(2025) Lun Audit Research No. (1178)]. All participants provided written informed consent before data collection. Audio recordings and transcripts were anonymised and stored securely. Only authorized members of the research team had access to the data.

### 2.6 Trustworthiness

Trustworthiness was ensured through several strategies. Credibility was enhanced by purposive sampling, in-depth interviews, and repeated reading of the transcripts. Dependability was supported by maintaining a clear description of the research process and discussing coding and theme development within the research team. Confirmability was strengthened through verbatim transcription, the use of participants' quotations, and reflexive discussions among the researchers during analysis. Reflexive discussions were conducted to examine how the RAM influenced coding and interpretation and to reduce the risk of forcing participants' accounts into predefined categories. Transferability was supported by providing detailed descriptions of the participants, study setting, and findings.

## 3 Results

A total of 12 participants were interviewed, and the age of the participants was 43.25 ± 8.23 years old; among them, one participant was unmarried but had a regular partner, and one participant was informed of a divorced status at the time of the interview. The basic characteristics of the participants are shown in Table 2. As shown in Table 3, findings were organized within the four predetermined adaptive modes of the RAM. Within these categories, eight subthemes were identified from participants' accounts. For clarity of presentation, each RAM mode was represented by a theme that summarized the main content of participants' experiences in that category: physical manifestations, sense of control, partner relationship, and sexual guidance.

**Table 2:** The general characteristics of the participants (n = 12).

NO.	Age	Educational level	Marital status	Number of stents	Number of PCIs	Complication
P1	52	High school	Married	1	1	Hypertension
P2	52	Primary school	Divorced	1	1	Heart failure, Renal insufficiency, Hypertension
P3	55	Junior high school	Married	3	2	Diabetes mellitus
P4	36	University	Married	1	1	None
P5	47	High school	Married	3	1	None
P6	43	University	Married	1	1	None
P7	45	High school	Married	1	1	None
P8	49	University	Married	2	1	Diabetes mellitus
P9	35	University	Unmarried with regular partner	1	1	None
P10	40	High school	Married	1	1	None
P11	28	University	Married	3	2	None
P12	37	University	Married	2	1	None

**Table 3:** Theme analysis.

RAM categories	Themes	Subthemes
Physiological mode	Physical manifestations	Rare discomfort symptoms
Self-concept mode	Sense of control	Worrying about recurrence
		Shifting to planned and careful sex
		Rebuilding a sense of control through self-management
Role-function mode	Partner relationship	Relationship patterns
		Supportive adaptation
Interdependence mode	Sexual guidance	No clear guidance
		Need for private, professional support

### 3.1 Physical manifestations

The theme of physical manifestations corresponded primarily to the physiological mode, as it concerned how participants perceived and responded to bodily changes, physical limitations, and functional readiness in relation to sexual activity after PCI.

#### 3.1.1 Rare discomfort symptoms

Regarding the timing of resumption, most participants began to gradually attempt sexual activity about 1–3 months after surgery. Most participants reported that sexual activity following PCI rarely directly triggers discomfort such as chest pain, chest tightness, or palpitations. They acknowledged its relatively low physical burden, providing a physiological foundation for resuming sexual activity.

*P3: “Not yet; I haven't experienced such symptoms (chest tightness, chest pain, etc.).”*

A very small number of participants reported potential physiological discomfort in scenarios that might increase cardiac load.

*P4: “After the heart attack (PCI), excessive or vigorous movements, especially in hot weather like now, might cause some discomfort (chest tightness).”*

Furthermore, we found that respondents with poor cardiac function would be affected by their poor physical condition, which would diminish their confidence and interest in sexual activities.

*P2: “Now when I go up the stairs, I have to take a break halfway; otherwise, I would be out of breath and wouldn't dare to think about such things.”*

### 3.2 Sense of control

The theme of sense of control corresponded primarily to the self-concept mode, as it reflected participants' psychological appraisal of sexual recovery and their efforts at emotional and behavioral self-regulation.

#### 3.2.1 Worrying about recurrence

Worry, and in some cases fear, about recurrent cardiac events represents the most prominent psychological burden. While medical interventions like stent implantation address physiological issues, they also create persistent psychological associations, causing participants to remain constantly anxious about their cardiac condition. This type of pressure persists even when one feels “in good health” following surgery.

*P6: “Even after my surgery, though it's been quite some time and I feel relatively well, I still can't help but worry—will this myocardial infarction recur? After all, there's something inside my heart. ”*

Additionally, participants grapple with multiple derivative anxieties, including self-doubt about diminished sexual function and concerns about potential medical complications. These complex emotions intertwine, collectively diminishing patients' initiative and satisfaction in their sexual lives.

*P1: “I heard contrast agents can harm kidney function. I've had two or three angiograms—I wonder if that's affecting my body.”*

Such vague concerns about medications and contrast agents further intensify psychological stress.

*P7: “Honestly, most of my worries stem from myself. I'm concerned about my own state—it's just mental apprehension.”*

#### 3.2.2 Shifting to planned and careful sex

In response to perceived multiple risks, participants spontaneously developed a series of preventive self-regulation behaviors. Primarily through reducing sexual activity frequency, lowering movement intensity, and shortening duration, they proactively avoided potential risks, trading the frequency and quality of sexual activity for psychological security.

*P9: “Try to exercise a bit more restraint; I used to feel more at ease before.”*

*P6: “Even after the treatment, I still think it's important to be careful about this aspect of life. We need to be mindful of the timing for sexual activity. ”*

#### 3.2.3 Rebuilding a sense of control through self-management

Some participants actively explore pathways to restore physical fitness and sexual function, attempting to improve their physical condition, though the scientific validity of their strategies varies significantly. A few participants opt for scientifically grounded approaches.

*P7: ”At first, there were some difficulties, but I gradually adapted—including through conditioning, medication, and exercise. Now these challenges have diminished.“*

Others rely on empirical dietary therapies or folk remedies.

*P2: ”I slaughtered eight pigeons alone. I bought ginseng to stew with them. I think as long as I can walk normally (without getting winded climbing stairs), I'll be okay. “*

### 3.3 Partner relationship

The theme of partner relationship corresponded primarily to the role-function mode, as it concerned changes in expectations, responsibilities, and interaction patterns within intimate relationships after PCI.

#### 3.3.1 Relationship patterns

Most participants reported that their partner relationship remained largely stable after PCI. Although sexual activity and physical function had changed, their partners' attitudes toward intimacy and the relationship itself were generally unchanged.

*P5: ”Physically, my partner's reaction to me hasn't changed much.“*

*P4: ”Before and after the surgery, my wife hasn't changed in this aspect (sexual life). Everything's still fine.“*

Some participants perceived subtle changes in the relationship after surgery, particularly in emotional closeness. These changes were usually described as mild rather than profound.

*P6: “Our marital relationship was somewhat affected. There were subtle changes—it just didn't feel as close anymore.”*

In very few cases, participants reported marked relationship strain or dissolution. Such accounts reflected broader changes in the partner relationship after illness, rather than being limited to sexual concerns alone.

*P2: “I can't do any physical labor now. Think about it—what else can I do? She resents me. She left. We ended up in court getting divorced. ”*

#### 3.3.2 Supportive adaptation

Many participants described their partners as becoming more understanding and supportive after the cardiac event. This support was reflected in greater emotional consideration and increased attention to the participant's health and psychological state.

*P10: “I feel like she's more considerate of my feelings now.”*

*P6: “Before, my partner didn't know [about my illness], and during intimacy she would complain that I wasn't the same as before, but since the heart attack diagnosis, she says taking care of my health is the most important thing—she's become much more understanding in that regard.”*

Participants also described adaptation through communication and modifications in intimacy. Open discussion between partners helped reduce misunderstanding and supported adjustment to changes after surgery. Some couples also adapted by shifting attention from sexual performance to other forms of emotional and physical closeness.

*P7: “My partner takes good care of me, and we communicate well.”*

*P9: “I would have been quite worried that my male persona had weakened, but my wife seemed to have noticed my self-doubt. She became more considerate and even started communicating with me. Gradually, I felt that everything was okay.”*

### 3.4 Sexual guidance

The theme of sexual guidance corresponded primarily to the interdependence mode, as it highlighted the extent to which sexual recovery was shaped by access to supportive relationships, professional input, and meaningful external guidance.

#### 3.4.1 No clear guidance

Participants face a dual challenge in accessing post-operative sexual health knowledge: a lack of professional guidance and limitations of non-professional channels, resulting in systemic knowledge gaps and cognitive confusion. First, influenced by traditional cultural taboos surrounding sexuality, significant communication barriers exist between doctors and patients: healthcare providers do not incorporate sexual health education into routine rehabilitation guidance, while participants rarely initiate consultations due to feelings of shame.

*P11: “I don't really know much about this area, and I haven't asked the doctors about the professional stuff either.”*

Most participants turn to non-professional channels like the internet and social media for information. However, these sources have significant limitations: content is disorganized, opinions are contradictory, and some information lacks scientific basis—even exaggerating risks—further deepening patients' confusion.

*P1: “Anyway, I saw on my phone that if either person feels discomfort during that time, you should stop immediately.”*

*P6: “Honestly, I've looked things up on Baidu and Douyin too, but one source says one thing and another says something else—it's hard to tell what's accurate. I've also seen reports about many online doctors lacking qualifications or exaggerating conditions just to attract traffic.”*

This dual dilemma makes it difficult for participants to develop scientifically grounded sexual health knowledge, creating a significant cognitive barrier to restoring sexual function.

#### 3.4.2 Need for private, professional support

Based on knowledge gaps and cognitive confusion, participants demonstrate clear and diverse demands for professional health support. Regarding core knowledge needs, participants are most concerned about the safety boundaries of post-operative sexual activity.

*P2, P6: “There's still a need to strengthen education in this area (timing, frequency, precautions, risks, etc.). Even though it's been a while since my surgery and I feel fine, I still have some worries. It's possible to avoid certain risks.”*

Regarding educational formats, participants generally prefer private, accessible resources that can be reviewed repeatedly, aligning with cultural expectations for privacy in sexual health discussions. Written materials, pamphlets, and online courses are most favored.

*P4, P5: “For convenience, providing pamphlets to take home is ideal—it's also more discreet.”*

Regarding service models, participants expect healthcare providers to shift from passive response to proactive engagement, formally integrating sexual health education into cardiac rehabilitation systems. They hope providers will proactively address related needs at critical junctures like discharge counseling and follow-up appointments, while offering accessible consultation channels such as scheduled one-on-one consultations and online Q&A platforms.

*P6: ”It should be tailored to the individual. I think the hospital (doctors/nurses) know me best as a patient and can provide guidance.“*

*P7: ”This is necessary.“ After all, it's often difficult to broach the subject. If you proactively intervene, that would be great. ”*

### 3.5 Integrative interpretation using the RAM

Using directed thematic analysis guided by the RAM, the four themes were interpreted in relation to key stimuli and adaptive responses across the physiological, self-concept, role-function, and interdependence modes. Table 4 summarizes the key stimuli identified in each theme, participants' adaptive responses, and basis for assigning data to the RAM category.

**Table 4:** Integrative summary of findings using the RAM.

RAM categories	Theme	Key stimuli identified	Adaptive responses	Basis for assigning data to the RAM category
Physiological mode	Physical manifestations	Poor physical condition, reduced stamina, dyspnoea, fatigue, and exertional or sexual activity-related discomfort	Gradual resumption of sexual activity; adjusted according to physical condition	Centered on bodily symptoms, functional capacity, physical tolerance for sexual activity
Self-concept mode	Sense of control	Fear of cardiac recurrence; uncertainty about the safety of sexual activity after PCI; concerns about stents, contrast agents, medication effects, and sexual function	Planned and cautious sexual activity; reduced frequency, intensity, or duration; self-monitoring and self-management to regain control	Centered on illness appraisal, perceived cardiac vulnerability, sexual confidence, emotional regulation, and reconstruction of control
Role-function mode	Partner relationship	Altered sexual and spousal role expectations; changes in sexual activity; mismatched partner responses; changes in relationship closeness	Partner communication; emotional support; adjustment of sexual expectations; distancing or reduced intimacy in strained relationships	Centered on changes in sexual and spousal role expectations, intimacy-related responsibilities, and partner interaction patterns
Interdependence mode	Sexual guidance	Limited access to private, professional, and individualized counseling; embarrassment in initiating sexual discussions; fragmented or inconsistent online information	Internet-based information seeking; withholding questions from professionals; desire for private, professional, and individualized support	Centered on access to supportive resources, communication with healthcare professionals, information seeking, and unmet needs for private guidance

## 4 Discussion

The findings suggest that sexual recovery after PCI was not determined solely by physical symptoms. Rather, it involved a multidimensional adaptation process shaped by perceived physical readiness, fear of recurrence, attempts to regain control, adjustment of partner roles, and access to reliable sexual health guidance. Although most participants reported few direct discomfort symptoms during sexual activity, many adopted cautious self-regulation strategies because of uncertainty about safety and insufficient professional counseling. Similar concerns were reported in a recent qualitative study of Iranian men with type 2 diabetes-related erectile dysfunction, where shame and embarrassment, disruption of sexual identity, threats to marital relationships, and inadequate sexual healthcare services were identified as key challenges (22). This convergence across different chronic diseases and cultural contexts strengthens the transferability of the present findings and highlights the importance of addressing psychological distress, relationship concerns, disclosure barriers, and unmet needs for professional support in men's sexual health care.

From the perspective of the physiological mode, most participants reported few obvious discomfort symptoms, during sexual activity in the months following PCI. This finding is consistent with evidence that sexual activity in clinically stable cardiac patients is generally comparable to mild-to-moderate physical exertion and causes only transient cardiovascular responses (9, 21). Current guidelines also support the recovery of sexual activity in clinically stable patients after appropriate assessment, rather than imposing broad restrictions solely due to coronary disease or PCI (6, 23). Moreover, early resumption of sexual activity within the first few months after acute myocardial infarction has been associated with better long-term survival (24). However, a few participants reported discomfort or avoidance in situations that increased cardiac workload, such as vigorous movement, hot weather, or poor physical capacity. Within the RAM, these experiences reflect physiological adaptation to bodily stimuli: adequate physical function facilitated sexual resumption, whereas dyspnoea, fatigue, or reduced exercise tolerance led to temporary postponement or avoidance. Overall, the limited occurrence of direct physical discomfort suggests that barriers to sexual recovery cannot be explained by physiological symptoms alone.

In the self-concept mode, participants' experiences reflected a threatened and gradually reconstructed sense of control. Although most reported few physical discomfort during sexual activity, many remained concerned about recurrent myocardial infarction, cardiac instability, stents, contrast agents, medication effects, or possible decline in sexual function. These concerns reflected participants' uncertainty about the bodily changes and the safety boundaries of sexual activity after PCI (15). By making participants question whether their post-PCI bodies were stable, safe, and capable of sexual activity, this uncertainty shaped their perceptions and beliefs about themselves rather than simply indicating physical limitation. Within the self-concept mode, it can therefore be understood as a psychological stimulus influencing their sense of bodily vulnerability, sexual capacity, and control. To regain a sense of safety, participants adopted planned and careful sex, including reducing frequency or intensity, shortening duration. These behaviors represented self-regulatory strategies rather than merely physiological limitation or sexual dysfunction (25). Such regulation may be adaptive when based on accurate information, but excessive risk perception or lack of professional counseling may lead to unnecessary restriction and reduced sexual confidence. Participants also attempted to rebuild control through broader self-management, such as medication adherence, exercise, and dietary adjustment. Evidence-based strategies may support both cardiovascular recovery and sexual adaptation (23), whereas reliance on folk remedies or unverified practices suggests an unmet need for reliable professional guidance.

In the role-function mode, sexual recovery involved adjustment of sexual and spousal roles within intimate relationships. Although partner relationship also reflected interdependence because partners served as important sources of emotional support, participants' accounts primarily emphasized changes in expectations, responsibilities, intimacy, and role performance. Most participants described stable or supportive relationships, in which partners showed understanding, adjusted sexual expectations, and communicated more openly. Such support helped participants reinterpret temporary changes in sexual activity as part of recovery rather than as personal or role failure. In contrast, a small number of participants reported emotional distancing or broader relationship strain, suggesting that insufficient communication and mismatched expectations may intensify role stress after PCI.

Deficiencies in the interdependence mode were most evident in participants' interactions with healthcare professionals and information resources (26). Participants reported limited access to professional, private, and individualized sexual counseling, while cultural embarrassment often prevented them from initiating discussions. In line with previous Chinese surveys (14), almost all participants in this study reported a lack of accurate sexual knowledge and very limited access to professional counseling. Participants expressed a clear need for guidance, yet the rates of proactive enquiry by clinicians and of formal sex education were extremely low, and appear even more worrisome than reports from Western countries such as the United States and Australia. When authoritative information is absent or ambiguous, patients may rely on fragmented online sources, which can reinforce confusion and misperceptions. Despite guideline recommendations that sexual health should be addressed as part of recovery (6, 23).

Therefore, we propose the following practical implications. First, healthcare professionals should strengthen their knowledge and skills related to sexual education. Sexual health care should become a routine, evidence-based, and culturally appropriate component of post-PCI rehabilitation (7, 27). In the Chinese cultural context, written materials, private consultation rooms, and secure online question-and-answer channels may be particularly useful. Second, a brief discharge or follow-up checklist could be considered, covering exercise tolerance, chest symptoms, fatigue, erectile function, medication use, fear of disease recurrence, misconceptions about stents or antiplatelet therapy, and partner overprotection (27). For clinically stable patients, professionals should provide clear advice on the gradual resumption of sexual activity, warning signs, and when to seek medical review. Medication-related sexual concerns should also be addressed (7). In this study, fear, perceived cardiac fragility, and uncertainty were more prominent than direct physiological symptoms. Brief partner counseling sessions may be provided during rehabilitation or follow-up to relieve concerns, correct exaggerated risk perceptions, reduce partner overprotection, and develop a gradual plan for restoring intimacy (28). For patients who are clinically stable but have strong fear, brief cognitive-behavioral strategies, such as risk explanation, relaxation training, graded exposure, and symptom-monitoring plans, may help rebuild body confidence and sexual self-efficacy.

This study has several limitations. The sample was small (*n* = 12), and all participants were recruited from a single tertiary hospital in Zhejiang Province, China, which may limit the transferability of the findings beyond young and middle-aged men in this setting. Experiences of sexual recovery may vary among women, older patients, and people in different healthcare or sociocultural contexts. In addition, because sexuality is a sensitive topic, self-reported interview data may have been influenced by recall bias or social desirability bias. Member checking, whereby findings are returned to participants for validation, was not conducted. Future studies should include more diverse populations, across sex, age, region, and cultural background.

## 5 Conclusions

This study found that, among young and middle-aged men after PCI, sexual recovery involved multidimensional adaptation across physical, psychological, relational, and informational domains. Worry about recurrence, perceived cardiac fragility, uncertainty, partner responses, and lack of professional guidance shaped their experiences and coping strategies. The RAM was useful for identifying key adaptive modes, interpreting relevant stimuli and responses, and explaining both effective and ineffective adaptation during sexual recovery. Future research should include more diverse populations and develop culturally appropriate, partner-centered sexual health education and rehabilitation interventions to support safe and confident sexual recovery after PCI.

## Data Availability

The original contributions presented in the study are included in the article/Supplementary material, further inquiries can be directed to the corresponding author.

## References

[1] Saito Y Kobayashi Y. Advances in technology and technique in percutaneous coronary intervention: a clinical review. Intern Med. (2026) 65:1–10. doi: 10.2169/internalmedicine.4505-24 39343561 PMC12854948

[2] Epifanio MS La Grutta S Alfano P Marcantonio S Piombo MA Ammirata M . Sexual satisfaction and quality of life in cardiovascular patients: the mediating role of anxiety. Healthcare. (2023) 11:290. doi: 10.3390/healthcare11030290 36766865 PMC9913900

[3] Neiman A Ginde S Earing MG Bartz PJ Cohen S. The prevalence of sexual dysfunction and its association with quality of life in adults with congenital heart disease. Int J Cardiol. (2017) 228:953–7. doi: 10.1016/j.ijcard.2016.11.192 27912205

[4] Lindau ST Abramsohn E Bueno H D'Onofrio G Lichtman JH Lorenze NP . Sexual activity and function in the year after an acute myocardial infarction among younger women and men in the United States and Spain. JAMA Cardiol. (2016) 1:754–64. doi: 10.1001/jamacardio.2016.2362 27579897 PMC5459405

[5] Abbasi A Ebrahimi H Bagheri H Basirinezhad MH Mirhosseini S Mohammadpourhodki R. A randomized trial of the effect of peer education on the sexual quality of life in patients with myocardial infarction. J Complement Integr Med. (2020) 17:20190204. doi: 10.1515/jcim-2019-0204 32701480

[6] O'Gara PT Kushner FG Ascheim DD Casey DEJ Chung MK de Lemos JA . 2013 ACCF/AHA guideline for the management of ST-elevation myocardial infarction: a report of the American College of Cardiology Foundation/American Heart Association Task Force on Practice Guidelines. Circulation. (2013) 127:e362–425. doi: 10.1161/CIR.0b013e3182742cf6 23247304

[7] Virani SS Newby LK Arnold SV Bittner V Brewer LC Demeter SH . 2023 AHA/ACC/ACCP/ASPC/NLA/PCNA guideline for the management of patients with chronic coronary disease: a report of the American Heart Association/American College of Cardiology Joint Committee on Clinical Practice Guidelines. J Am Coll Cardiol. (2023) 82:833–955. 37480922 10.1016/j.jacc.2023.04.003

[8] Je M. Sexual activity as a trigger for cardiovascular events: what is the risk? Am J Cardiol. (1999) 84:2N−5N. doi: 10.1016/S0002-9149(99)00218-0 10503569

[9] Piegza M Smolarczyk J Piegza J. Sexual and cardiovascular healthFactors influencing on the quality of sexual life of coronary heart disease patients - a narrative review. Vasc Health Risk Manag. (2025) 21:51–60. doi: 10.2147/VHRM.S484566 39931042 PMC11807849

[10] Ebrahim S May M Ben Shlomo Y McCarron P Frankel S Yarnell J . Sexual intercourse and risk of ischaemic stroke and coronary heart disease: the Caerphilly study. J Epidemiol Community Health. (2002) 56:99–102. doi: 10.1136/jech.56.2.99 11812807 PMC1732071

[11] Brandis Kepler S Hasin T Benyamini Y Goldbourt U Gerber Y. Frequency of sexual activity and long-term survival after acute myocardial infarction. Am J Med. (2020) 133:100–7. doi: 10.1016/j.amjmed.2019.06.019 31295439

[12] Mangolian Shahrbabaki P Mehdipour-Rabori R Gazestani T Forouzi MA. Iranian nurses' perspective of barriers to sexual counseling for patients with myocardial infarction. BMC Nurs. (2021) 20:196. doi: 10.1186/s12912-021-00697-x 34641868 PMC8513282

[13] Hyde EK Martin DE Rieger KL. Factors shaping the provision of sexual health education for adults with acute coronary syndrome: a scoping review. Patient Educ Couns. (2020) 103:877–87. doi: 10.1016/j.pec.2019.11.017 31767244

[14] Zhang HY Kong XP Wu H Shen YZ. Investigation of sexual health education status for patients with coronary heart disease resuming sexual life. Chin. Nurs. Manag. (2011) 11:60–3.

[15] Wang H Xu HF. Study on health needs for resuming sexual life after PCI in young and middle-aged patients with coronary heart disease. J Nurs Manag. (2021) 21:63–6.

[16] Rahim L Allana S Steinke EE Ali F Khan AH. Level of knowledge among cardiac nurses regarding sexual counseling of post-MI patients in three tertiary care hospitals in Pakistan. Heart Lung. (2017) 46:412–6. doi: 10.1016/j.hrtlng.2017.09.002 28988654

[17] Lilly K Walsh AL Foreman R Moran C Taylor J. Communicating about sexual activity and intimacy after a heart attack: a cross-sectional survey of Australian health professionals. Eur J Cardiovasc Nurs. (2024) 23:478–85. doi: 10.1093/eurjcn/zvad110 38170837

[18] Wang X Wang LZ. Investigation of sexual life status in young and middle-aged male patients with coronary heart disease before and after stent implantation. J Nurs. (2007) 11:7–10. doi: 10.16460/j.issn1008-9969.2007.11.042

[19] DeSanto-Madeya S Fawcett J. Toward understanding and measuring adaptation level in the context of the Roy adaptation model. Nurs Sci Q. (2009) 22:355–9. doi: 10.1177/0894318409344753 19858515

[20] Stewart M. Qualitative inquiry: perceptions of sexuality by African Americans experiencing haemodialysis. J Adv Nurs. (2013) 69:1704–13. doi: 10.1111/jan.12028 23046384

[21] Gagliardi BA. The experience of sexuality for individuals living with multiple sclerosis. J Clin Nurs. (2003) 12:571–8. doi: 10.1046/j.1365-2702.2003.00739.x 12790871

[22] Rozveh AK Afshari A Sayadi L Mohajeri Tehrani MR Mehrnoush V Khazaei A. Exploring sexual health challenges in men with type 2 diabetes-related erectile dysfunction: a qualitative study. BMC Public Health. (2026) 26:954. doi: 10.1186/s12889-026-26640-w 41692712 PMC13011304

[23] Steinke EE Jaarsma T Barnason SA Byrne M Doherty S Dougherty CM . Sexual counseling for individuals with cardiovascular disease and their partners: a consensus document from the American Heart Association and the ESC Council on Cardiovascular Nursing and Allied Professions (CCNAP). Circulation. (2013) 128:2075–96. doi: 10.1161/CIR.0b013e31829c2e53 23897867

[24] Cohen G Nevo D Hasin T Benyamini Y Goldbourt U Gerber Y. Resumption of sexual activity after acute myocardial infarction and long-term survival. Eur J Prev Cardiol. (2022) 29:304–11. doi: 10.1093/eurjpc/zwaa011 33624045

[25] Steinke EE Jaarsma T. Sexual counseling and cardiovascular disease: practical approaches. Asian J Androl. (2015) 17:32–9. doi: 10.4103/1008-682X.135982 25219908 PMC4291873

[26] Lindau ST Abramsohn E Gosch K Wroblewski K Spatz ES Chan PS . Patterns and loss of sexual activity in the year following hospitalization for acute myocardial infarction (a United States National Multisite Observational Study). Am J Cardiol. (2012) 109:1439–44. doi: 10.1016/j.amjcard.2012.01.355 22546209 PMC3341956

[27] Inayat S Aziz F Younas A Durante A. Determinants of sexual health and sexual quality of life after cardiovascular surgeries: an integrative review. Heart Lung Circulation. (2024) 33:1414–26. doi: 10.1016/j.hlc.2024.05.010 38969608

[28] Barrett OEC Ho AK Finlay KA. Sexual function and sexual satisfaction following spinal cord injury: an interpretative phenomenological analysis of partner experiences. Disabil Rehabil. (2024) 46:86–95. doi: 10.1080/09638288.2022.2159073 36576221

