# Supplementary material for: Experience of sexual recovery after percutaneous coronary intervention in young and middle-aged men: a qualitative study
Source: Front Public Health. 2026 Aug 13;14:1876557. doi: 10.3389/fpubh.2026.1876557 (PMC13518333; doi:10.3389/fpubh.2026.1876557)
Supplement: Supplementary file 1 [file Supplementary_File_1.docx]

Supplementary Material

1 **Consolidated criteria for reporting qualitative studies (COREQ): 32-item checklist**

Developed from:

Tong, A., Sainsbury, P., & Craig, J. (2007). Consolidated criteria for reporting qualitative research (COREQ): A 32-item checklist for interviews and focus groups. *International Journal for Quality in Health Care, 19*(6), 349–357. https://doi.org/10.1093/intqhc/mzm042

| **No. Item** | **Guide questions/description** | **Answer** | **Reported on Page #** |
| --- | --- | --- | --- |
| Domain 1: Research team and reﬂexivity |  |  |  |
| Personal Characteristics |  |  |  |
| 1.Interviewer*/*facilitator | Which author*/*s conducted the interview or focus group? | MY | Sections 2.3  Data collection |
| 2. Credentials | What were the researcher’s credentials? E.g. PhD, MD | MY held a Master’s degree in nursing; ZY held a Master’s degree in nursing; HDX held a Bachelor’s degree in nursing. | Sections 2.2 Participants and recruitment;  Sections 2.3 Data collection |
| 3. Occupation | What was their occupation at the time of the study? | MY was a male manager; ZY and HDX were nurses. | Sections 2.2 Participants and recruitment;  Sections 2.3 Data collection |
| 4. Gender | Was the researcher male or female? | The interviewer (MY) was male. | Sections 2.3 Data collection |
| 5.Experiencing and training | What experience or training did the researcher have? | All researchers have received training in qualitative research methods | Sections 2.3 Data collection |
| Relationship with participants |  |  |  |
| 6. Relationship established | Was a relationship established prior to study commencement? | MY was not involved in participants’ routine clinical care. The recruiting nurses (ZY and HDX) were not responsible for the participants’ current clinical care at the time of recruitment, and some participants had not been directly cared for by them. | Sections 2.2 Participants and recruitment;  Sections 2.3 Data collection |
| 7. Participant knowledge of the interviewer | What did the participants know about the researcher? e.g. personal goals, reasons for doing the research | MY was not involved in participants’ routine clinical care.  The recruiting nurses (ZY and HDX) were not responsible for the participants’ current clinical care at the time of recruitment, and some participants had not been directly cared for by them. | Sections 2.2 Participants and recruitment;  Sections 2.3 Data collection |
| 8.Interviewer characteristics | What characteristics were reported about the interviewer/facilitator? e.g. Bias, assumptions, reasons and interests in the research topic | We avoided making assumptions before collecting and analyzing the interview data. Reflexive discussions were conducted during analysis to minimise bias and assumptions. | Section 2.6 Trustworthiness |
| Doman 2: study design |  |  |  |
| Theoretical framework |  |  |  |
| 9.Methodological orientation and Theory | What methodological orientation was stated to underpin the study? e.g. grounded theory, discourse analysis, ethnography, phenomenology, content analysis | Qualitative descriptive design using directed thematic analysis, informed by the Roy Adaptation Model. | Section 2.1 Design;  Section 2.4 Data analysis |
| Participant selection |  |  |  |
| 10.Sampling | How were participants selected? e.g. purposive, convenience, consecutive, snowball | Purposive sampling. | Sections 2.2 Participants and recruitment |
| 11.Method of approach | How were participants approached? e.g. face-to-face, telephone, mail, email | Eligible individuals were contacted by telephone after identification through the electronic medical record system. | Sections 2.2 Participants and recruitment |
| 12.Sample size | How many participants were in the study? | 12 participants | Sections 2.2 Participants and recruitment;  Sections 3 Results |
| 13. Non-participation | How many people refused to participate or dropped out? Reasons? | All invited participants completed the study. No participants withdrew. | Sections 2.3 Data collection |
| Setting |  |  |  |
| 14.Setting of data collection | Where was the data collected? e.g. home, clinic, workplace | Interviews were conducted in a private room at the hospital after routine follow-up visits. | Sections 2.2 Participants and recruitment |
| 15. Presence of non-participants | Was anyone else present besides the participants and researchers? | No | Sections 2.2 Participants and recruitment |
| 16.Description of sample | What are the important characteristics of the sample? *e.g. demographic data, date* | Demographic and clinical characteristics are reported in Table 2. | Table 2 |
| Data collection |  |  |  |
| 17.Interview guide | Were questions, prompts, guides provided by the authors? Was it pilot tested? | Yes. | Table 1 |
| 18. Repeat interviews | Were repeat interviews carried out? If yes, how many? | No | N/A |
| 19.Audio/visual recording | Did the research use audio or visual recording to collect the data? | Yes. Audio recording | Sections 2.3 Data collection |
| 20.Field notes | Were ﬁeld notes made during and/or after the interview or focus group? | Yes | Sections 2.3 Data collection |
| 21. Duration | What was the duration of the interviews or focus group? | About 20-80 mins each | Sections 2.3 Data collection |
| 22. Data saturation | Was data saturation discussed? | Yes | Sections 2.3 Data collection |
| 23. Transcripts returned | Were transcripts returned to participants for comment and/or correction? | No | Section 4 Discussion |
| Domain 3: analysis and fingings |  |  |  |
| Data analysis |  |  |  |
| 24. Number of data coders | How many data coders coded the data? | 2 coders (ZY and HDX) | Section 2.4 Data analysis |
| 25.Description of the coding tree | Did authors provide a description of the coding tree? | No | N/A |
| 26. Derivation of themes | Were themes identiﬁed in advance or derived from the data? | Top-level categories are theory-driven, whereas lower-order codes and subthemes are data-driven. | Sections 2.3 Data collection;  Section 2.4 Data analysis |
| 27. Software | What software, if applicable, was used to manage the data? | NVivo 15 | Section 2.4 Data analysis |
| 28.Participant checking | Did participants provide feedback on the ﬁndings? | No | Section 4 Discussion |
| Reporting |  |  |  |
| 29. Quotations presented | Were participant quotations presented to illustrate the themes */* ﬁndings? Was each quotation identidied? E.g. participant number | Yes. | Section 3 Results |
| 30. Data and findings consistent | Was there consistency between the data presented and the ﬁndings? | Yes | Section 3 Results |
| 31.Clarity of major themes | Were major themes clearly presented in the ﬁndings? | Yes | Section 3 Results |
| 32.Clarity of minor themes | Is there a description of diverse cases or discussion of minor themes? | Yes | Section 3 Results |

N/A: not applicable.
